# SoyOD: An Integrated Soybean Multi-omics Database for Mining Genes and Biological Research

**DOI:** 10.1093/gpbjnl/qzae080

**Published:** 2024-11-13

**Authors:** Jie Li, Qingyang Ni, Guangqi He, Jiale Huang, Haoyu Chao, Sida Li, Ming Chen, Guoyu Hu, James Whelan, Huixia Shou

**Affiliations:** State Key Laboratory of Plant Environmental Resilience, College of Life Sciences, Zhejiang University, Hangzhou 310058, China; The Provincial International Science and Technology Cooperation Base on Engineering Biology, International Campus of Zhejiang University, Haining 314400, China; State Key Laboratory of Plant Environmental Resilience, College of Life Sciences, Zhejiang University, Hangzhou 310058, China; State Key Laboratory of Plant Environmental Resilience, College of Life Sciences, Zhejiang University, Hangzhou 310058, China; State Key Laboratory of Plant Environmental Resilience, College of Life Sciences, Zhejiang University, Hangzhou 310058, China; State Key Laboratory of Plant Environmental Resilience, College of Life Sciences, Zhejiang University, Hangzhou 310058, China; State Key Laboratory of Plant Environmental Resilience, College of Life Sciences, Zhejiang University, Hangzhou 310058, China; State Key Laboratory of Plant Environmental Resilience, College of Life Sciences, Zhejiang University, Hangzhou 310058, China; The Provincial International Science and Technology Cooperation Base on Engineering Biology, International Campus of Zhejiang University, Haining 314400, China; Crop Research Institute, Anhui Academy of Agricultural Sciences, Hefei 230000, China; State Key Laboratory of Plant Environmental Resilience, College of Life Sciences, Zhejiang University, Hangzhou 310058, China; The Provincial International Science and Technology Cooperation Base on Engineering Biology, International Campus of Zhejiang University, Haining 314400, China; State Key Laboratory of Plant Environmental Resilience, College of Life Sciences, Zhejiang University, Hangzhou 310058, China; The Provincial International Science and Technology Cooperation Base on Engineering Biology, International Campus of Zhejiang University, Haining 314400, China

**Keywords:** Soybean, Database, Genome, Transcriptome, Phenome

## Abstract

Soybean is a globally important crop for food, feed, oil, and nitrogen fixation. A variety of multi-omics studies have been carried out, generating datasets ranging from genotype to phenotype. In order to efficiently utilize these data for basic and applied research, a soybean multi-omics database with extensive data coverage and comprehensive data analysis tools was established. The Soybean Omics Database (SoyOD) integrates important new datasets with existing public datasets to form the most comprehensive collection of soybean multi-omics information. Compared to existing soybean databases, SoyOD incorporates an extensive collection of novel data derived from the deep-sequencing of 984 germplasms, 162 novel transcriptomic datasets from seeds at different developmental stages, 53 phenotypic datasets, and more than 2500 phenotypic images. In addition, SoyOD integrates existing data resources, including 59 assembled genomes, genetic variation data from 3904 soybean accessions, 225 sets of phenotypic data, and 1097 transcriptomic sequences covering 507 different tissues and treatment conditions. Moreover, SoyOD can be used to mine candidate genes for important agronomic traits, as shown in a case study on plant height. Additionally, powerful analytical and easy-to-use toolkits enable users to easily access the available multi-omics datasets, and to rapidly search genotypic and phenotypic data in a particular germplasm. The novelty, comprehensiveness, and user-friendly features of SoyOD make it a valuable resource for soybean molecular breeding and biological research. SoyOD is publicly accessible at https://bis.zju.edu.cn/soyod.

## Introduction

Soybean [*Glycine max* (L.) Merr.] is an annual herbaceous crop in the legume family. It is an important source of plant-derived oil, protein, and biologically fixed nitrogen. Soybean originated in China and has been cultivated for more than 5000 years. Cultivated soybean is domesticated from wild soybean (*Glycine soja* Sieb. and Zucc.) and is now a global agronomic crop [1]. Over time, cultivated varieties with a range of desired agronomic traits have been developed to suit human needs. However, there are increasing global demands for soybeans, with China alone importing more than 80 million tons annually for food, feed, and industrial uses. These demands underscore the need for developing new soybean varieties with improved yield, quality, and stress tolerance to unfavourable and changing environmental conditions. Since the release of the first soybean reference genome from the variety Williams 82 in 2010 [2], significant progress has been made in soybean genomics research. To date, researchers have successfully assembled the chromosomal-level genomes of more than 50 diverse soybean varieties [3,4]. Several complete gap-free genomes (telomere-to-telomere, T2T) have been published [5–9]. Accompanying this are extensive datasets on genetic variation from thousands of soybean accessions and other omics datasets, including genome resequencing, pan-genomes, transcriptomes, phenomes and more. These resources have greatly accelerated soybean functional genomics research and molecular breeding [10].

To integrate multi-omics data, several soybean multi-omics databases have been established, such as Soybase [11], SoyKB [12], SoyOmics [13], and SoyMD [14]. These omics platforms function as comprehensive repositories for genomic, genetic, and related data resources concerning soybean. However, no single database integrates datasets in a timely manner, which hinders the efficiency of data utilization and application to soybean research. Additionally, omics analyses frequently depend on specific reference genomes, which limits their applicability to other genomes. Providing easy-to-use tools to facilitate conversion between different genome coordinates is crucial. To address these issues, we developed a comprehensive multi-omics database structured on the available soybean datasets to provide an intuitive interface in a user-friendly, one-stop platform supported by online toolkits for functional gene mining in soybean.

The newly developed multi-omics database encompasses deep-sequencing data from 984 accessions, transcriptomic data across seed development in different varieties, extensive phenotypic data for genome-wide association study (GWAS), and phenotypic images from our laboratory. It integrates a wide range of omics datasets, including six modules “Genome, Phenome, Variome, Population, Transcriptome, and Synteny”, thus enabling rapid searching of genotypic and phenotypic data across germplasm or gene information. The database is named Soybean Omics Database (SoyOD) and is available at https://bis.zju.edu.cn/soyod. The primary objective of developing this database and toolkit is to facilitate soybean functional studies and accelerate breeding efforts.

## Database content and features

### Overview of SoyOD interface

**Figure 1:**
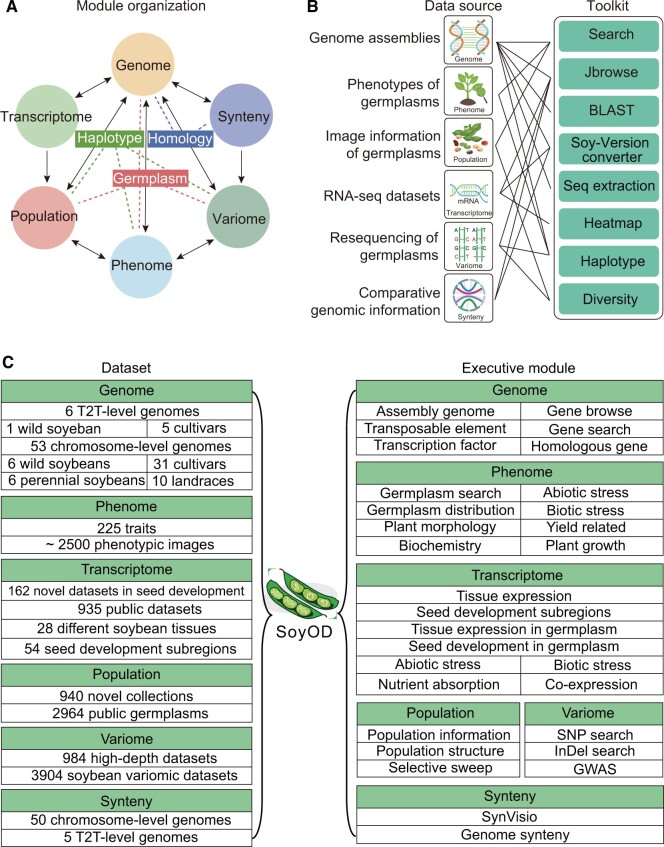
Overview of data sources, toolkits, and module interactions of SoyOD **A**. Module framework of SoyOD, including genome, phenome, variome, population, transcriptome, and synteny modules. The arrow indicates that the module is interacted with by another module. The dotted lines indicate that the haplotype, homology, and germplasm are composed of different modules. **B**. The interactive interfaces among data sources and toolkits facilitate efficient data exchange and analysis. A connection line shows that this segment of data can be linked or integrated with another segment. **C**. Description of the multi-omics datasets in the six executive modules (left) as well as the submodules of these executive modules (right). SoyOD, Soybean Omics Database; T2T, telomere-to-telomere; SNP, single nucleotide polymorphism; InDel, insertion/deletion; GWAS, genome-wide association study.

To establish a comprehensive repository of soybean multi-omics data, we meticulously integrated datasets from multiple sources to establish the soybean multi-omics database known as SoyOD. The SoyOD homepage provides an overview of features, module introduction, available tools, latest news, and updates log. The top menu is a multi-omics search tool allowing users to input gene ID, chromosome region, genome name, germplasm name, or phenotype of interest for the retrieval of information from the database. The detailed information of a gene can be found by viewing a related gene ID, including a basic description, gene structure and sequence, available quantitative trait loci (QTLs), and predicted functional categories. We have developed six interactive modules, namely “Genome”, “Phenome”, “Population”, “Transcriptome”, “Variome”, and “Synteny”, to integrate all datasets (**Figure 1**A). The analysis toolkits ensure interactive uses among the different modules (Figure 1B). The homepage provides drop-down menus and data summaries of the six modules, as well as links to “Toolkits”, “Download”, and “About”. SoyOD have collected datasets from the last 20 years of global high-throughput sequencing projects, allowing all data to be accessed through a unified platform.

In the genome module, we collected 59 published soybean genome assemblies, which consisted of 6 perennial *Glycine* spp., 47 chromosome-level genomes (including 6 wild soybeans, 31 cultivars, and 10 landraces), and 6 T2T-level genomes (including 1 wild soybean and 5 cultivars) (Figure 1; Table S1). The genome module includes several submodules, namely “Assembly genome”, “Gene browse”, “Gene search”, “Transcription factor”, “Transposable element”, and “Homologous gene”, which can be easily searched using gene ID or chromosome region (Figure 1C).

The phenome module contains 398,485 records of 225 phenotypes, including those related to plant morphology, yield related, plant growth, biochemistry, abiotic stress, and biotic stress (Table S2). The phenome module also contains phenotypic data for 4097 soybean germplasm resources and approximately 2500 phenotypic images. These phenotypic data and images could be used for verification of a particular germplasm. Users can find the relevant phenotypic information and images according to a variety name or ID number search.

Within the transcriptome module, a total of 1097 RNA sequencing (RNA-seq) libraries, including 162 datasets from our laboratory and 935 from public databases, were mapped to the corresponding genomes, and gene expression profiles were generated (Figure 1C). Following classification, these datasets were organized into eight submodules, including “Tissue expression”, “Seed development subregions”, “Tissue expression in germplasm”, “Seed development in germplasm”, “Abiotic stress”, “Biotic stress”, “Nutrient absorption”, and “Co-expression” (Figure 1C; Table S3). The transcriptomic data can be mapped to five reference genomes, including the two most recent T2T-level genomes (Williams 82 T2T and Zhonghuang 13 T2T) and three widely used chromosome-level genomes (Williams 82 v2, Williams 82 v4, and Zhonghuang 13 v2). Users can conveniently retrieve gene expression data by entering the corresponding gene ID. All transcriptomic raw data were downloaded and compared to multiple different assembled genomes to obtain read count, fragments per kilobase of exon model per million mapped fragments (FPKM), and transcripts per kilobase of exon model per million mapped reads (TPM) values. For more intuitive browsing of gene expression patterns, an electronic fluorescent pictograph (eFP) browser was developed for visualizing gene expression levels from different dataset submodules. Additionally, a heatmap toolkit is available for users to customize the expression matrix and generate personalized heatmaps. This function facilitates efficient access to gene expression data, enhancing the overall user experience. The co-expression network was constructed by the weighted correlation network analysis (WGCNA) package in R using datasets generated by different organizations and integrated into the co-expression submodule. Users can input one or more gene IDs into this submodule to investigate the co-expression network associated with specific genes of interest. Overall, the transcriptome module offers a comprehensive set of features and tools that enable users to explore the level of gene expression through an intuitive interface.

The population module combines the data from 940 newly sequenced accessions with 44 public datasets for a total of 984 deep-sequencing datasets with an average depth of 43×. In order to increase the population scales, 2920 additional public accessions were integrated, resulting in a total of 3904 population datasets (Figure 1C; Table S4). The deep-resequencing datasets can be browsed or searched either separately or with the published resequencing datasets. Furthermore, the selective sweep analysis serves as a foundation for users to identify genes associated with domestication and crop improvement.

The variome module gathered comprehensive variation data by resequencing a diverse array of germplasm resources. The variome module includes a total of 984 deep-resequencing datasets (Figure 1C; Table S4). All resequencing data were compared to the Zhonghuang 13 v2 reference genome. The deep-resequencing of 984 accessions resulted in a total of 5,685,352 single nucleotide polymorphism (SNPs) and 1,361,946 insertions/deletions (InDels). Additionally, public resequencing datasets were analyzed and integrated, comprising a total of 3904 accessions, generating 7,193,573 SNPs and 753,361 InDels (Table S4). Users can query variation information within a specified chromosome region. The variome module also includes GWAS analysis, which will be elaborated in greater detail below.

In the synteny module, we performed comparative genomic analysis of 55 assembled genomes (Figure 1C). Users can readily find structural variation (SV) information through comparison with the Zhonghuang 13 v2 reference genome. In addition, the platform incorporates a feature for quick browsing of genomic collinearity, utilizing the SynVisio web service (https://synvisio.github.io/). User can select different chromosomes to browse by synteny and dot plot views. Meanwhile, users can quickly generate comparative genomic variation data by conducting searches across specified chromosome regions using the genome variation search modules.

### Germplasm resources and phenotypes

**Figure 2:**
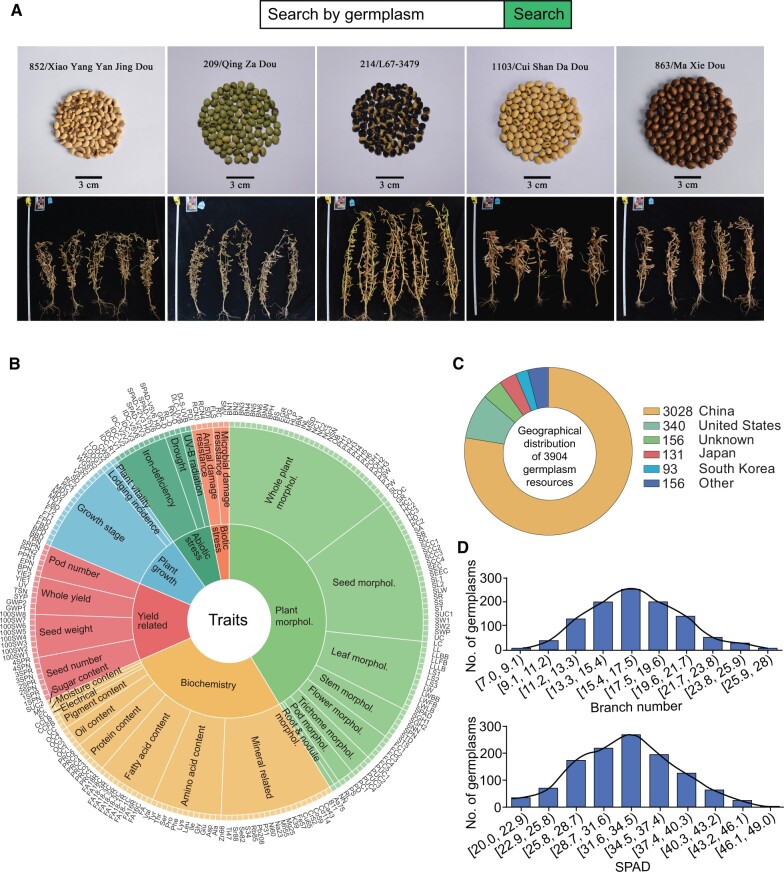
Phenotypic data retrieval module for soybean germplasm resources **A**. Search bar and image gallery for soybean germplasm resources. **B**. Sunburst chart of 225 traits. **C**. The geographical distribution of 3904 germplasm resources. **D**. Distribution of branch number (upper) and SPAD value (lower). morphol., morphology; UTR, untranslated region; CDS, coding sequence; SPAD, soil and plant analyzer development.

Germplasm resources are essential for crop improvement, biodiversity conservation, and research in plant genetics and breeding. These resources encompass a broad range of genetic materials from plants, including seeds, tissue cultures, DNA, and other plant parts used for breeding new varieties or studying genetic traits. The SoyOD database includes phenotypic data of 4097 soybean germplasm resources and approximately 2500 phenotypic images. Users can retrieve the germplasm data using name/ID of a germplasm or the trait of interest. The database provides detailed information for each germplasm, including phenotypic images and trait measurements (**Figure 2**A).

A phenotype is an observable, physical characteristic of an organism. In the context of germplasm resources, phenotypic information is crucial for understanding how different genetic materials react in various environments. The phenotypic data provide important information for breeding programs aimed at achieving desirable traits, such as high yield, disease resistance, abiotic stress tolerance, and others. In SoyOD, information pertaining to the germplasm origin and phenotypes was integrated into the phenome module. Phenotypic data were collected for 225 different traits, including 53 traits from this study and 172 traits from several other public sources. The 225 traits are classified into a three-level catalogue, and users can select a phenotype by interacting with the sunburst graph (Figure 2B). The phenome module is divided into eight sub-modules (Germplasm search, Germplasm distribution, Plant morphology, Biochemistry, Plant growth, Yield related, Abiotic stress, and Biotic stress). The database contains a diverse array of germplasm resources from all over the world, including 3028 varieties from China (Figure 2C; Table S4). The distributions of two traits, namely branch number (ranging from 7 to 28 branches) and soil and plant analyzer development (SPAD) value (representing relative chlorophyll content, ranging from 22 to 55) were plotted as examples (Figure 2D). The germplasm interface facilitates rapid searches of accessions. It allows users to explore their characteristics with corresponding images, avoiding confusion between different varieties.

### Consolidation and interrogation of genomic data

In the genome module, we collected the 59 published soybean genomes, including 6 perennial *Glycine* spp., 47 chromosome-level genomes, and 6 T2T-level genomes (Table S1). The summary data on all 59 genome assemblies, including genome size, type, source, assembly level, and gene density, were integrated into the genome module. Complete annotation information was unavailable for four genomes, which were consequently excluded from the partial analysis.

The mRNA, coding sequence (CDS), and protein sequence annotations are included in the gene browser, with each Gene ID linked to its gene structure, associated QTLs, GO, KEGG and PFAM predictions. Users can query and download these datasets using a gene ID search. Within the submodules, users can browse transcription factors (TFs) and transposons by assembly or by family name. Homologous genes can be browsed by gene ID or homologous group. The 73,270 groups were derived by comparing the predicted protein sequences from each genome. Meanwhile, comparative genomic analysis between 55 genomes (excluding four genomes without annotation information) and the Zhonghuang 13 v2 reference genome was conducted to generate synteny alignments and structural variation information, which are available in the synteny module.

### Population genetic variation detection and GWAS

**Figure 3:**
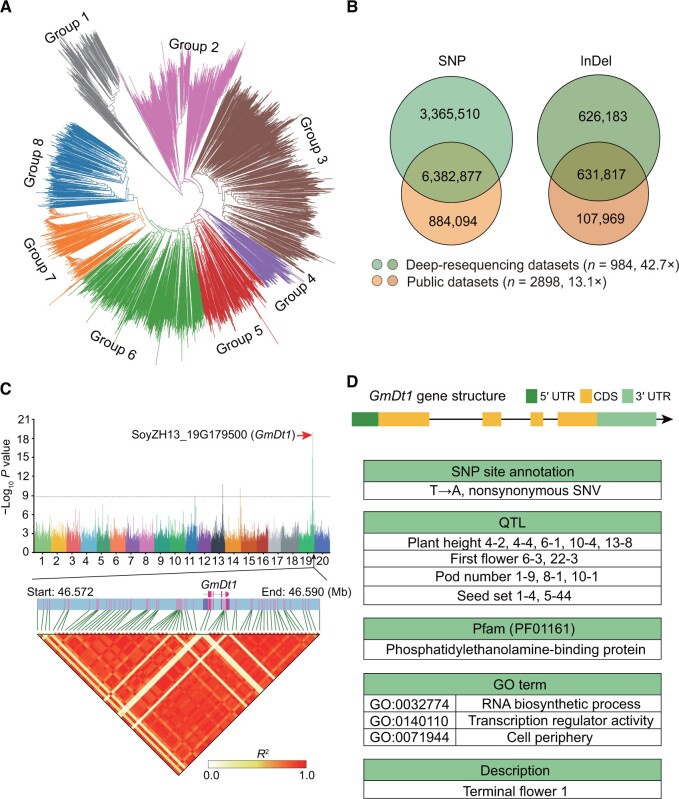
Gene mining via GWAS **A**. Phylogenetic tree of 3904 germplasm resources. **B**. The number of SNPs and InDels detected by deep-resequencing (42.7×) from 984 accessions and 2898 public datasets (13.1×) using MAF < 1%, miss rate > 10% as filtering criteria. **C**. GWAS analysis of plant height using EMMAX method as a case study. The LDblock displays information about linkage disequilibrium between 46.572 Mb and 46.590 Mb on chromosome 19, using a dataset of 984 resequenced samples. **D**. The *GmDt1* gene structure, SNP site annotation, QTLs, Pfam domains, and GO terms retrieved from SoyOD. MAF, minor allele frequency; EMMAX, efficient mixed model association expedited; SNV, single nucleotide variant; QTL, quantitative trait locus; GO, Gene Ontology.

A phylogenetic tree was constructed using 3904 accessions, categorizing them into eight distinct clusters, with the wild populations primarily concentrated in group 1 (**Figure 3**A). By comparing the SNPs and InDels between the 984 deep-resequencing datasets and the 2898 published datasets [15], multiple alleles and different alleles at the same variation site were removed, revealing a large number of shared SNPs (*n* = 6,382,877) and InDels (*n* = 631,817). Interestingly, there were 3,365,510 SNPs and 626,183 InDels that were only detected in the 984 deep-sequencing datasets, suggesting that increased sequencing depth improves the detection of genetic variants (Figure 3B).

GWAS analysis using the 984 resequencing datasets was conducted on 24 traits, including many related to plant morphology and yield. The GWAS submodule can be used to search for variations related to certain traits. We found a region significantly associated with plant height (−log_10_  *P* value > 8.8) on chromosome 19, which we hypothesized contained a dominant gene controlling plant height in soybean. This chromosome region contained a total of 849 loci. Through further screening, a significant locus was identified with an exon at Chr19:46582743, with an allele that leads to a nonsynonymous substitution (Figure 3C). The linkage disequilibrium (LD) block under the haplotype tool also showed a strong linkage between this position and the preceding position. Search for the related gene in the main database search bar, which is consistently located at the top of arbitrary page. We found that this site matches the gene SoyZH13_19G179500 (*GmDt1*), which encodes a protein “Terminal flower 1” (Figure 3D). Through the gene ID search functionality, QTL information can be filtered to highlight regions associated with plant height, such as QTLs plant height 4-2, 4-4, 6-1, 10-4, and 13-8. *Dt1* was known to regulate stem growth habit and flowering in soybean [16]. The GWAS analysis in this study reidentified *GmDT1*, confirming the efficacy of the resequencing data and analysis methods for discovering novel genes.

### Gene functional exploration and expression pattern visualization

**Figure 4:**
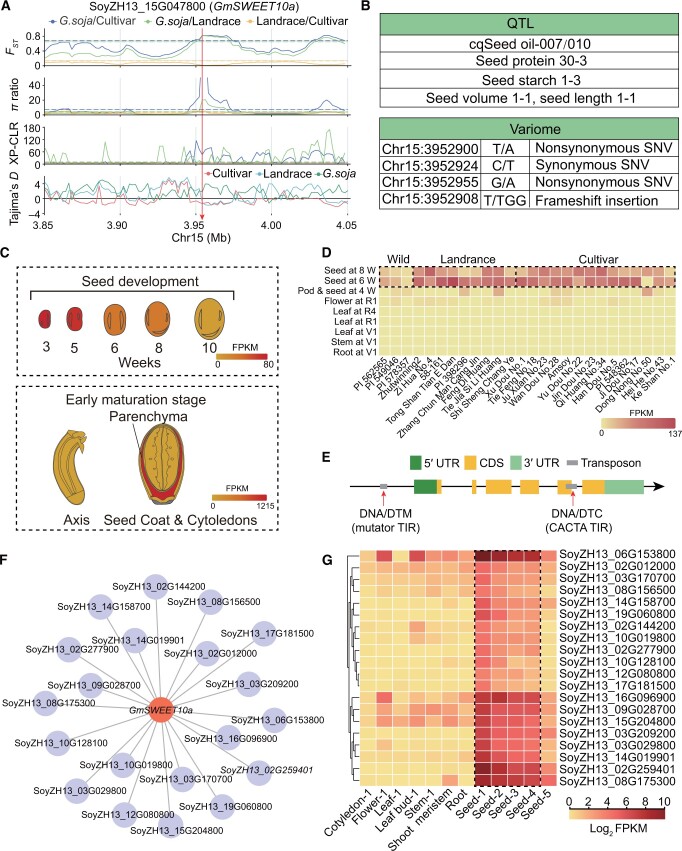
A case study characterizes *GmSWEET10a* using SoyOD **A**. Genetic variations (*F_ST_*, *π* ratio, XP-CLR, and Tajima’s *D*) were calculated across the 0.2-Mb genomic region of the SoyZH13_15G047800 locus using genomic resequencing data from 984 accessions, including *G. soja*, landraces, and cultivars. 20-kb sliding windows with 2-kb steps were used. **B**. *GmSWEET10a* QTL and variation information retrieved by the gene search submodule. **C**. Expression of the *GmSWEET10a* gene in the eFP viewer in the tissue expression and seed development submodules. **D**. Tissue expression in different germplasms. W indicates weeks. **E**. Transposon information for the *GmSWEET10a* gene, including both the gene body and the 2000-bp region upstream of its transcription start site, using the transposable element module of the Williams 82 genome. **F**. WGCNA for *GmSWEET10a*. **G**. Expression profile of the co-expressed genes of *GmSWEET10a*. eFP, electronic fluorescent pictograph; FPKM, fragments per kilobase of exon model per million mapped fragments; WGCNA, weighted gene co-expression network analysis; DTM, DNA transposon mutator; DTC, DNA transposon CACTA; TIR, terminal inverted repeat.

The SoyOD platform utilizes multi-omics approach to analyze population selection and visualize gene expression patterns, enhancing our understanding of complex biological phenomena. Here we present a comprehensive framework demonstrating how to perform soybean population selection analysis using an integrated method and visualize gene expression using an eFP browser or heatmap tools. *GmSWEET10a* has been reported to regulate soybean oil and protein content as well as seed size [17]. We use the SoyOD website to further illustrate the important role of *GmSWEET10a* in soybean seed development (**Figure 4**). For the case study, the population selective sweep submodule was used to view the data for Chr15 in the 3.85–4.05 Mb region. Four selective sweep models, namely the neutrality test statistics of fixation index (*F_ST_*), Tajima’s *D* statistic (Tajima’s *D*), cross-population composite likelihood ratio index (XP-CLR), and nucleotide polymorphism ratio (*π* ratio), indicate that the gene region was under strong artificial selection during soybean domestication (Figure 4A). In addition, the QTLs cqSeed oil-007/010, seed protein 30-3, seed starch 1-3, seed volume 1-1, and seed length 1-1 were linked to this region (Figure 4B). These QTL descriptions are consistent with previous studies of the phenotypes [17]. In the coding region, four genetic variants were found, including two nonsynonymous substitutions and one frameshift insertion, which may alter the sequence or structure of the SWEET protein (Figure 4B) and are therefore worthy of further investigation.

The transcriptomic data can be searched by selecting the Gene ID and corresponding genome. By searching the Zhonghuang 13 v2 genome with SoyZH13_15G047800 (*GmSWEET10a*) and visualizing with the eFP browser, the user can readily observe high expression levels in early development stages (Figure 4C). Choosing the “seed development subregions” submodule, we found that *GmSWEET10a* was predominantly expressed in the seed coat parenchyma at the early maturation stage (Figure 4C), underscoring its potential role in shaping seed morphology. Moreover, when exploring the expression of *GmSWEET10a* in the “tissue expression in germplasm” submodule, an intriguing pattern was evident in the heatmap. It was found that *GmSWEET10a* was specifically expressed in seeds, with higher levels in landraces and cultivated accessions compared to the wild soybean accessions (Figure 4D). The differential expression suggests that this gene may have been under selective pressure during domestication. Meanwhile, two DNA transposons within the genome region of *GmSWEET10a* were identified, located in the upstream and fifth exon regions of the gene, which may affect the function of the encoded protein (Figure 4E).

To identify genes that are co-expressed with *GmSWEET10a*, we performed co-expression analysis under the transcriptome module. Twenty genes were associated with *GmSWEET10a* (Figure 4F). After downloading the list of co-expressed genes, we used the heatmap (under the Toolkits) to visualize the expression information of the 20 genes. These genes are selectively expressed during the early seed stage, with several showing high expression levels (Figure 4G). They are potential candidates for further functional analysis to explore gene interactions.

### Selective sweep analyses and novel gene discovery with SoyOD

**Figure 5:**
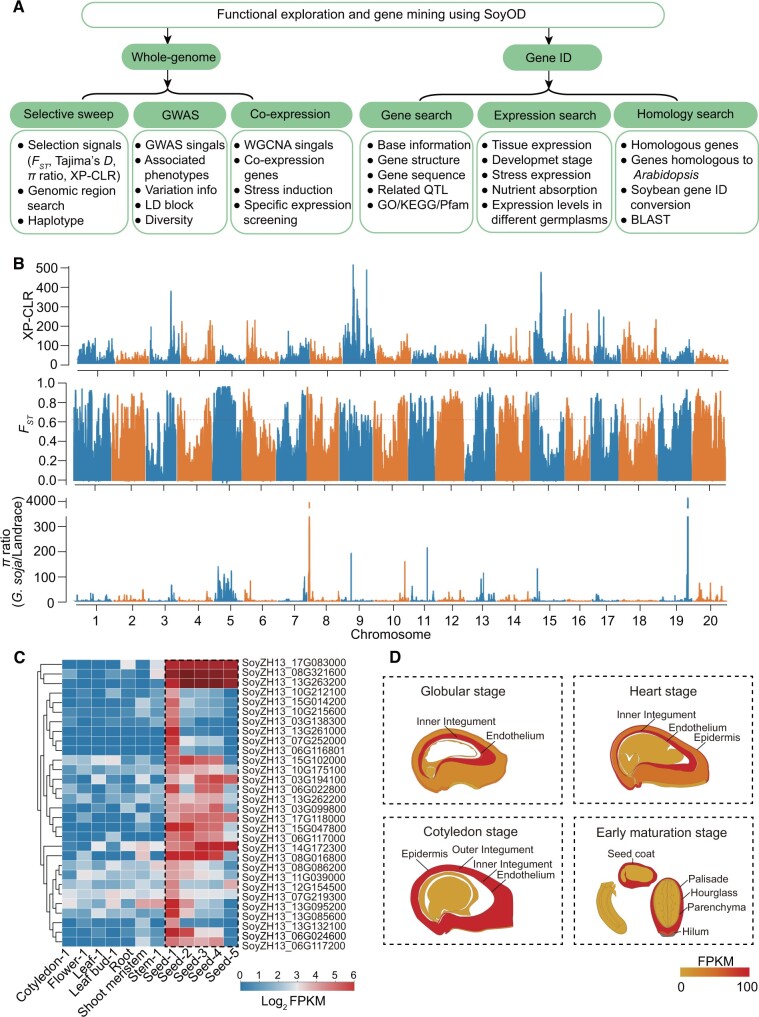
Discovery of new genes through SoyOD database exploration **A**. The technical flow diagram for exploring and mining novel genes using the SoyOD database. **B**. Selection signals were analyzed using XP-CLR, *F_ST_*, and *π* ratio to assess regions under selection during soybean domestication (from wild soybean to landrace). The red dashed horizontal lines represent the genome-wide significant threshold for selection signals. **C**. The expression patterns of domestication-related genes were analyzed across different tissues. **D**. The expression level of the SoyZH13_06G024600 gene during early seed development was analyzed. Data were extracted from Zhonghuang 13 v2 reference genome.

SoyOD can be used to explore and mine novel genes, and the technical flow diagram is summarized (**Figure 5**A). Users can access selective sweeps, GWAS, and co-expression data on SoyOD to search for genes of a target trait (Figure 5A). SoyOD contains a process for mining important genes based on population selection and expression data (Figure 5A). The population selection data rely on three measures, *F_ST_*, *π* ratio, and XP-CLR, and show that soybean contains 1411 genes selected during domestication (Figure 5B; Table S5) and 75 genes selected during improvement (Table S6), with 3 genes in both sweeps. We combined these domestication genes with the tissue expression module to search for domestication genes expressed in specific regions of seeds. The 30 relevant genes were shown using the Heatmap toolkit (Figure 5C). We selected the example gene, SoyZH13_06G024600, for viewing in the seed development eFP module, and found that it was specifically expressed in the seed coat (Figure 5D).

Through the homology module, a user can find homologous genes within soybean and *Arabidopsis* genomes, access their functional descriptions, or conduct BLAST search to find related information by target gene sequence (Figure 5A). A search using a gene ID will return target gene information including Gene Ontology (GO), Kyoto Encyclopedia of Genes and Genomes (KEGG), Pfam, QTL, gene sequence, gene structure data, and gene expression profiles (Figure 5A). Once a user identifies a gene of interest, they can use the gene ID to infer gene function.

### Online omics interactive tool

We developed the toolkit portal on SoyOD to meet the growing demand for integrated multi-omics studies. SoyOD hosts a diverse array of online analysis tools, including JBrowse, BLAST, Soy-Version converter, Seq extraction, Heatmap, Diversity, and Haplotype (Figure 1). With access to sequences and genes from 55 published soybean genomes (excluding four genomes without annotations), the interactive tools on SoyOD significantly reduce the time researchers must spend to navigate multiple databases. Users can conduct comprehensive analyses and query various genomes through a single, unified platform, eliminating the requirement for switching between disparate databases. This innovation not only enhances efficiency but also fosters deeper insights by simplifying access to a broad array of genomic information. JBrowse allows users to easily query genomic annotation, variation, and expression information, providing an overview of the data. The BLAST module can use an uploaded file to perform alignment of coding or protein sequences for cross-genome comparisons. Users can select one or more genomes and enter multiple sequences simultaneously for convenient and rapid identification of homologous sequences by BLAST analysis. To convert between datasets, we have developed the Soy-Version converter tool, which allows users to switch between different genome versions. With the sequence extraction tool, a user can search for gene sequence information by gene ID or by region of the chromosome. Users can effortlessly retrieve gene expression information using the heatmap tools by entering multiple gene IDs and selecting the corresponding expression data. Additionally, they have the option to customize the output heatmap to display specific matrix information. Personalization of the heatmap can be used to adjust its maximum and minimum values, as well as modifying its color scheme. The population diversity and haplotype tools allow users to better explore whether a gene has undergone selective sweeps.

## Discussion

Cultivating new soybean varieties to enhance yield and quality is crucial for meeting the global demands of the soybean market. Functional genomics and molecular breeding in soybean increasingly rely on multi-omics analysis based on existing datasets [18–20]. To support genetic improvement and molecular breeding of soybean, we integrated six modules, the Genome, Phenome, Variome, Population, Transcriptome, and Synteny into an easily usable, comprehensive multi-omics database named SoyOD. During this process, we included and compared multiple omics datasets generated over the past decade (**Table 1**). Compared with the available databases, SoyOD has several advantages: (1) SoyOD contains the largest and most up-to-date soybean omics datasets, including 59 assembly genome datasets, 398,485 records of 225 phenotypes, 1097 RNA-seq transcriptomes, genetic variation data from 3904 soybean accessions, selective sweep information, and 59 genome synteny and comparative genomic analysis. This makes SoyOD the most comprehensive resequencing soybean population ever studied. (2) SoyOD offers new deep-resequencing data from this study, which can be used for more precise genetic variation analysis. (3) SoyOD contains phenomic data of soybean germplasms, which is crucial for understanding genotype–phenotype relationships. (4) SoyOD implements multi-module interactive use, which greatly enhances the user-friendliness and intuitiveness of the interface. Users can input a gene ID to search the corresponding gene annotations, expression levels, homologous genes, related QTL information, *etc*. The comparison of SoyOD with other available soybean genome databases is listed in Table 1.

**Table 1:** Comparison between commonly used soybean databases and SoyOD

Database	No. of genome assemblies	No. of accessions	No. of RNA-seq libraries	No. of traits	No. of phenotype images	GWAS
SoyOD	59*	3904	1097	225	∼ 2500	√
Soybase	14	20,087^#^	–	126	–	–
SoyFGB	–	2214	–	42	–	√
SoyOmics	36	2898	342	115	28	√
SoybeanGDB	39	3379	146	–	–	–
SoyGVD	–	4414	–	48	–	√
SoyMD	38	24,501^#^	721	125	–	√

*Note*:

* indicates the inclusion of 6 T2T-level genomes;

^#^ indicates the inclusion of the SNP50k data; – indicates that the data are not included; √ indicates that the data are included.

With the accelerated development and application of sequencing technology in the post-genomic era, gene mining and molecular breeding increasingly rely on multi-omics analysis [20]. SoyOD integrates the majority of multi-omics research data into a user-friendly interface with a variety of online analysis tools. SoyOD is designed as a dynamic platform to integrate the latest global soybean multi-omics data. Notably, SoyOD is accessible online without requirement for registration, making it readily available to researchers. While we recommend the Chrome browser for the best browsing experience, the database is optimized to function across various browsers. In the future, we will continue to update this database and develop analytical methods and tools to make it an important resource for global soybean research.

## Materials and methods

### Data acquisition

To construct an accessible, comprehensive multi-omics database for soybean, we developed six interactive modules including “Genome”, “Phenome”, “Population”, “Transcriptome”, “Variome”, and “Synteny”. In the genome module, we collected data from 59 previously reported, high-quality assembled genomes, including 47 chromosome-level genomes [6 wild soybean (*Glycine soja*), 10 landraces, 31 improved cultivars], 6 T2T-level assemblies of the genomes from Zhonghuang 13 (including two versions namely T2T and T2T-2), Williams 82, Yundou1, Yesheng71, and Jack, and 6 recently published genomes from different perennial soybeans species (*G. cyrtoloba*, *G. dolichocarpa*, *G. falcata*, *G. stenophita*, *G. syndetika*, and *G. tomentella D3*) [2,5–9,15,21–27]. The publication for each genome is listed in Table S1. However, four genomes (Zhonghuang 13 T2T-2, ENREI, PI594527, and Zhe Nong 6) did not contain annotation information, and only partial genomic analyses were performed.

The phenome module collects 398,485 records of 225 phenotypes obtained from 53 local data resources and 172 public data resources over multiple years. All traits were divided into six different phenotypic categories, namely plant morphology, yield related, plant growth, biochemistry, abiotic stress, and biotic stress.

Transcriptomic data were collected from 1097 RNA-seq libraries from different tissues and conditions, including seed development stages, abiotic stress, biotic stress, and so on (Table S3). To determine the expression patterns of various soybean varieties throughout seed development, we sequenced 162 RNA-seq libraries. These libraries were derived from 54 different accessions, which were sampled at four key stages of seed development from 15 representative soybean germplasm resources. In addition, we downloaded and integrated 342 expression datasets from 28 tissues from cultivar Williams 82 [28], 27 tissues from cultivar Zhonghuang 13 [4], and 9 tissues from 26 other varieties [15]. We included data from 297 accessions of soybean seeds sampled from different stages of early seed development by laser capture microdissection [29,30]. In the abiotic stress submodule, 296 libraries were taken from plants subjected to different conditions, including exposure to salinity, drought, cold, temperature, flooding, dehydration, PEG 8000, submergence, pH, CO_2_, and auxin treatments. In the nutrient absorption submodule, treatments included nitrogen, phosphorous, potassium, iron, and zinc. References for these transcriptomic datasets are listed in Table S3.

In the variome and population modules, we combined 940 newly sequenced, and 44 published deep-resequencing accessions to construct high-quality genome variation maps based on second-generation sequencing and phenotypic data of representative germplasms. With the addition of 2920 public resequencing datasets [15,23,24], the total resequencing data included 3904 datasets (Table S4).

### Gene annotation and functional analysis

The 6 T2T-level and 53 chromosome-level genomes listed in Table S1 were integrated. After excluding four unannotated genomes, 55 genomes were annotated using eggNOG with default program settings, including the GO terms, KEGG pathways, Pfam domains, and gene descriptions [31]. Prediction of TFs was carried out using protein homology alignment to *Arabidopsis* or soybean TFs by the Plant TFDB [32]. Transposons in the 59 genomes were predicted using the EDTA pipeline [33] by detecting repeat sequences with the default parameters.

### Variation calling and annotation

The 984 deep-resequencing datasets were compared to the Zhonghuang 13 v2 reference genome [3] using the Burrows-Wheeler Aligner (BWA; v0.7.17-r1188) [34]. SAMtools (v1.14) [35] and Picard package (v2.27.5) were used to flag duplicated reads. The detection was performed using the Genome Analysis Toolkit (GATK; v4.2.2.0) HaplotypeCaller [36], genotyping was done with GenotypeGVCFs [36], and the separation of SNPs and InDels was performed using the GATK SelectVariants option [36]. Finally, low-quality SNPs and InDels were filtered using GATK VariantFiltration. SNP and InDel annotations were performed based on the Zhonghuang 13 v2 reference genome using the ANNOVAR package [37]. An additional 2898 resequencing data were downloaded from the National Genomics Data Center, as reported by Liu et al [15]. The public resequencing data were integrated, resulting in the module that encompasses variation data from 3904 accessions. For subsequent analysis, all data were uniformly filtered and annotated.

### Selective sweep and GWAS analyses

To analyze the population structure and construct an evolutionary tree, we screened a subset of SNPs and InDels using VCFtools (v0.1.16) [38]. The cutoff settings for the data from the 3904 resequenced accessions were missing rate > 10% and minor allele frequency (MAF) < 1%; for the 984 accessions, the SNP missing rate was > 10% and the MAF was < 5%, while the InDel missing rate was > 50% and the MAF was < 1%. The evolutionary trees were constructed using Phylip software (v3.697) with 1000 bootstraps. Population structure was analyzed using the admixture (v1.3.0) program with parameters ranging from *k* = 2 to *k* = 10 according to the phylogenetic analysis order. To analyze each subpopulation, we employed VCFtools and XP-CLR (updated based on Python) [39] to calculate the genetic diversity (*π*), XP-CLR, and *F_ST_* values using a window size of 20 kb and a step size of 2 kb across the entire soybean genome. Tajima’s *D* was calculated using a 2-kb window size. The top 5% value was chosen as the significance threshold.

GWAS was performed using the efficient mixed model association expedited (EMMAX) software [40]. For GWAS, the threshold was set at 0.01/(total number of SNPs), corresponding to −log_10_  *P* value = 8.8 as whole-genome significance cutoff. Manhattan and QQ plots were drawn by CMplot (R package). LDBlockShow was used to draw LD block heatmaps for a specified area using the default parameters [41].

### Comparative genomic analysis

The genomic sequences of Zhonghuang 13 v2 were compared with those of 55 other assemblies (excluding four unannotated ones) using the NUCmer program (v4.0.0rc1) within the MUMmer4 suite [42]. After filtering one-to-one alignments to retain only those with a minimum length of 100 bp (utilizing the delta-filter program from MUMmer with the specified parameters ‘-m -i 90 -l 100’), the show-coords program was used to convert the delta file into readable matching coordinates. Syri (v1.6.3) [43] was employed to extract the coordinates and features of structural variations. Finally, the plostr program from Syri was used to generate visual results. Gene synteny was identified using the Python-based McScan tool [44]. For visualization of the McScan results, the dotplots and synteny were drawn in SynVisio (https://vanilla-genome-tools.cirad.fr/synvisio/) before integration into SoyOD.

A total of 494,137 genes from the 55 soybean genome assemblies were utilized to construct gene clusters. All protein sequences were obtained, and homologies were detected using the OrthoFinder (v2.5.5) software (with the parameters ‘-S diamond -M msa -T fasttree’). Homologous genes were aggregated into clusters, yielding a total of 73,270 groups.

### Transcriptomic analysis

A total of 1097 transcriptomic libraries were downloaded using the prefetch (v2.8.0) software, and then adapter sequences and low-quality reads were removed using fastp (v0.12.4) [45] (Table S3). To obtain a comprehensive gene expression profile, we aligned datasets to five frequently-used reference genomes (Zhonghuang 13 v2, Zhonghuang 13 T2T, Williams 82 v2, Williams 82 v4, and Williams T2T), while other RNA-seq datasets were aligned to the corresponding genome (Table S1) using Hisat2 (v2.2.1) software with default parameters [46]. Gene expression levels were quantified to obtain count, FPKM, and TPM values using the ‘featureCounts’ R package [47]. The FPKM values were then analyzed by WGCNA [48] for co-expression analysis using the topological overlap matrix (TOM) approach. Expression values below 0.25 were excluded from the analysis. The resulting co-expression network was constructed by calculating the Pearson correlation coefficients between pairwise gene expression levels.

### Website construction and design

SoyOD (https://bis.zju.edu.cn/soyod) is a web-based application built on the Python Django (v2.2.14) framework for the backend and uses HTML, CSS, and jQuery (v3.4.1) as the frontend JavaScript framework, ensuring a powerful and flexible foundation for data processing and management. Data storage and retrieval are managed through an Apache 2 web server (v2.6.1) and uses MySQL (v8.0.35) as its database engine. The visualization software includes Echarts (v5.4) [49] for line, bar, and pie charts, as well as sunburst graphs. A JavaScript library, D3 (v6.7.0) [50], is used for evolutionary trees. JBrowse2 (v2.8.0) [51] serves as the genome browser. Diamond (v2.0.14) is used for gene sequence BLAST analysis. The entire system uses the Ubuntu operating system (v20.04; Canonical). Some of the icons were designed using Adobe Photoshop (v19.0) and Adobe Illustrator (v27.0). The database is accessible online without registration and is recommended for use in Google Chrome, Microsoft Edge, Mozilla Firefox, or Apple Safari. SoyOD employs this combination of technologies to create an intuitive and user-friendly interface, delivering a seamless experience for accessing soybean-related data and tools.

## Supplementary Material

qzae080_Supplementary_Data

## Data Availability

The raw RNA-seq data generated in this study have been deposited in the Genome Sequence Archive [52] at the National Genomics Data Center, Beijing Institute of Genomics, Chinese Academy of Sciences / China National Center for Bioinformation (GSA: CRA019036), and are publicly accessible at https://ngdc.cncb.ac.cn/gsa. SoyOD is publicly available at https://bis.zju.edu.cn/soyod.

## CRediT author statement

**Jie Li:** Conceptualization, Data curation, Formal analysis, Investigation, Methodology, Software, Validation, Visualization, Writing – original draft, Writing – review & editing. **Qingyang Ni:** Data curation, Formal analysis, Validation, Visualization, Methodology. **Guangqi He:** Data curation, Validation. **Jiale Huang:** Validation, Data curation. **Haoyu Chao:** Methodology. **Sida Li:** Data curation. **Ming Chen:** Conceptualization, Data curation, Project administration, Resources, Supervision, Writing – review & editing. **Guoyu Hu:** Resources, Investigation. **James Whelan:** Funding acquisition, Project administration, Writing – review & editing. **Huixia Shou:** Conceptualization, Data curation, Funding acquisition, Resources, Project administration, Supervision, Writing – review & editing. All authors have read and approved the final manuscript.
