# Supplementary material for: SoyOD: An Integrated Soybean Multi-omics Database for Mining Genes and Biological Research
Source: Genomics Proteomics Bioinformatics. 2024 Nov 13;22(6):qzae080. doi: 10.1093/gpbjnl/qzae080 (PMC11757165; doi:10.1093/gpbjnl/qzae080)
Supplement: qzae080_Supplementary_Data [file qzae080_supplementary_data.zip › Supplementary Materials captions.docx]

# Supplementary material

**Table S1 Chromosome- and T2T-level genome assemblies collected and used in SoyOD**

**Table S2 Summary of phenotypic datasets**

**Table S3 Summary of collected transcriptome datasets**

**Table S4 The 3904 re-sequenced accessions of *Glycine soja* and *Glycine max***

**Table S5 Screening of selective signals for domestication-related genes**

**Table S6 Screening of selective signals for improvement-related genes**
